# Protein S-nitrosylation: specificity and identification strategies in plants

**DOI:** 10.3389/fchem.2014.00114

**Published:** 2015-01-07

**Authors:** Olivier Lamotte, Jean B. Bertoldo, Angélique Besson-Bard, Claire Rosnoblet, Sébastien Aimé, Siham Hichami, Hernán Terenzi, David Wendehenne

**Affiliations:** ^1^CNRS, UMR 1347 AgroécologieDijon, France; ^2^ERL CNRS 6300Dijon, France; ^3^Departamento de Bioquímica Centro de Ciências Biológicas, Centro de Biologia Molecular Estrutural, Universidade Federal de Santa CatarinaFlorianópolis, Brasil; ^4^Université de Bourgogne, UMR 1347 AgroécologieDijon, France; ^5^Institut National de la Recherche Agronomique, UMR 1347 AgroécologieDijon, France

**Keywords:** nitric oxide, S-nitrosylation, post-translational modifications, plants, signaling, biotin switch technique

## Abstract

The role of nitric oxide (NO) as a major regulator of plant physiological functions has become increasingly evident. To further improve our understanding of its role, within the last few years plant biologists have begun to embrace the exciting opportunity of investigating protein S-nitrosylation, a major reversible NO-dependent post-translational modification (PTM) targeting specific Cys residues and widely studied in animals. Thanks to the development of dedicated proteomic approaches, in particular the use of the biotin switch technique (BST) combined with mass spectrometry, hundreds of plant protein candidates for S-nitrosylation have been identified. Functional studies focused on specific proteins provided preliminary comprehensive views of how this PTM impacts the structure and function of proteins and, more generally, of how NO might regulate biological plant processes. The aim of this review is to detail the basic principle of protein S-nitrosylation, to provide information on the biochemical and structural features of the S-nitrosylation sites and to describe the proteomic strategies adopted to investigate this PTM in plants. Limits of the current approaches and tomorrow's challenges are also discussed.

## Introduction

In animals, the free radical nitric oxide (NO) serves as an important messenger of intra- and extracellular pathways and regulates a myriad of physiological processes (Schmidt and Walter, 1994; Mustafa et al., 2009). For instance, its involvement as a modulator of vascular tone, neurotransmission, platelet aggregation, reproductive systems, and immune responses are widely recognized. The involvement of NO as a physiological mediator is not restricted to animals. Notably, results of intensive investigations achieved over the past 15 years indicate that NO also regulates diverse biological processes in plants. Key roles for NO have been demonstrated in seed dormancy, embryogenic cell formation, root development and gravitropic bending, flowering, stomatal closure, growth regulation of pollen tubes, nutrition and particularly iron homeostasis, immunity, and adaptive responses to various abiotic stresses (Wilson et al., 2008; Wendehenne et al., 2014; Yu et al., 2014). Comprehensive studies were undertaken in order to clarify the molecular mechanisms underlying NO functions in plants. Clearly, the cellular activities of NO are numerous and complex: NO operates through reactive oxygen species (ROS) and classical second messengers including Ca^2+^ and cGMP, regulates the activity of metabolic and signaling proteins such as protein kinases, impacts the organization of cytoskeleton and actin-dependent vesicle trafficking and modulates the expression of numerous genes involved in essentially all cellular functions (Besson-Bard et al., 2009; Kasprowicz et al., 2009; Leitner et al., 2009; Gaupels et al., 2011; Jeandroz et al., 2013; Trapet et al., 2014). Furthermore, cross-talks between NO and key hormones including auxin, abscisic acid, salicylic acid (SA), jasmonic acid, ethylene, and cytokinins have been reported (Lamattina et al., 2003; Wilson et al., 2008; Terrile et al., 2012; Feng et al., 2013; Mur et al., 2013). Another issue of investigations concerned the origins of NO produced by plant cells. Nitrite is unquestionably a main substrate for NO synthesis through both non-enzymatic and enzymatic processes involving nitrate reductase (Gupta et al., 2011). A body of arguments also suggests that plants could possess an enzyme which, similarly to animal nitric oxide synthases (NOS), could use L-arginine as a substrate (Corpas et al., 2009). This latter possibility is still debated.

How NO governs cellular reactions at the molecular level has been and is still the subject of important researches in animal biology. The current available data illustrate that NO actions, as well as those of certain NO-derived species, depend on chemical modifications of proteins. Three main processes are now recognized: nitration referring to the binding of a NO_2_ group to Tyr residues, metal- and S-nitrosylation referring to the binding of a NO group to a transition metal or a Cys residue, respectively (Mannick and Schonhoff, 2002). S-nitrosylation has emerged as an important NO-dependent post-translational modification (PTM) of proteins regulating a large variety of cellular functions and signaling events (Hess et al., 2005; Gould et al., 2013). In plants, the search and functional analysis of S-nitrosylated proteins has also grown substantially this last decade. These investigations resulted in original data and S-nitrosylation is now accepted as a cell signaling mechanism with important functional implication in various plant physiological processes (Lindermayr and Durner, 2009; Spadaro et al., 2010; Astier et al., 2012b). In the present review, we have considered the question of S-nitrosylation in plant cells, with a particular emphasis on proteomic approaches for the identification of S-nitrosylated proteins either by proteomic approaches or by algorithm-based SNO site identification.

## Basic knowledge about protein S-nitrosylation in animals

**Figure 1:**
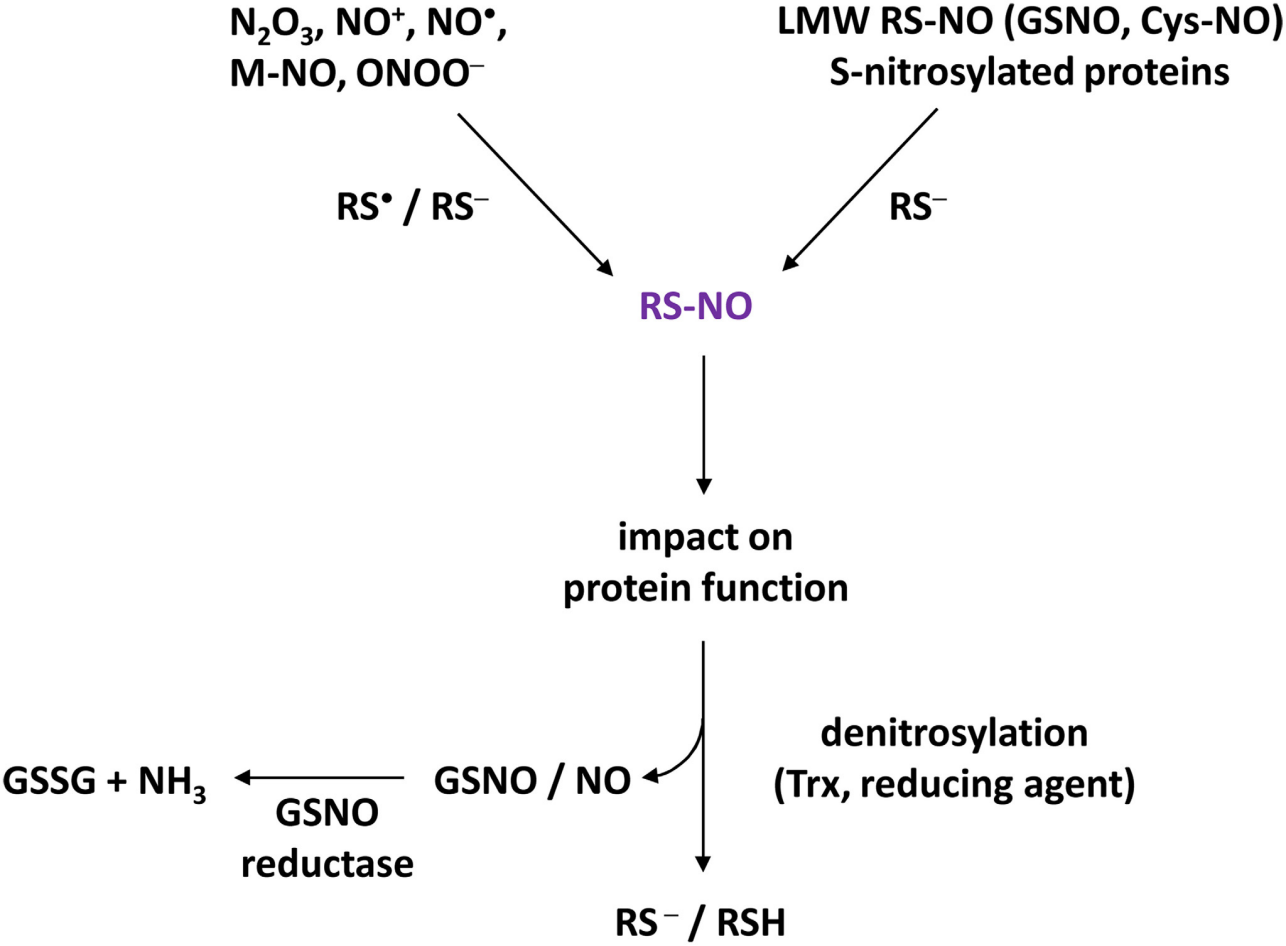
**Basic concept of S-nitrosylation**. Higher oxides of NO (such as N_2_O_3_, NO^+^), NO as a free radical (NO^•^), metal-NO complexes (M-NO) or NO derivatives such as peroxynitrite (ONOO^−^) are able to trigger S-nitrosylation by interacting with Cys thiolate (RS^−^) or thiyl (RS^•^) of target proteins, thus leading to S-nitrosothiols (RS-NO). Furthermore, S-nitrosylation can be mediated by transnitrosylation through S-nitrosylated proteins and low molecular weight (LMW) S-nitrosothiols such as nitrosoglutathione (GSNO) or S-nitrosocysteine (Cys-NO). S-nitrosylation impacts the function of target proteins by affecting their activities (activation, inhibition), their subcellular localizations and interactions with partners. S-nitrosylation is a reversible process mediated by reducing compounds such as GSH but also by thioredoxins (Trx). Denitrosylation could therefore lead to the formation of GSNO which, in turn, is metabolized into glutathione disulfide (GSSG) and ammonia by GSNO reductase (GSNOR). Therefore, Trx and GSNOR appear to be critical for SNO homeostasis.

S-nitrosylation is a reversible covalent chemical reaction in which a NO moiety is coupled to a critical Cys thiolate (-S^−^) on a target protein (Martinez-Ruiz et al., 2011; Gould et al., 2013). This process leads to the formation of an S-nitrosothiol (SNO) and is mediated by NO through a thiyl radical recombination by higher oxides of NO such as dinitrogen trioxide (N_2_O_3_) or nitrosonium cation (NO^+^), by metal-NO complexes or low molecular weight S-nitrosothiols including S-nitrosocysteine (CysNO) and the major physiological NO donor nitrosoglutathione (GSNO) (Hess et al., 2005; Hill et al., 2010; Smith and Marletta, 2012). Several S-nitrosylated proteins including SNO-hemoglobin, -thioredoxin (Trx), -caspase 3, or -glyceraldehyde-3-phosphate dehydrogenase (GAPDH) can also catalyze the transfer of their NO group to an adjacent thiol of a binding partner; a mechanism referred as transnitrosylation (Figure 1) (Kornberg et al., 2010; Nakamura and Lipton, 2013; Sengupta and Holmgren, 2013). This PTM triggers conformational changes of proteins, can affect their activities, sub-cellular localization and interactions with partners. Over the past 20 years, thanks to the emergence of dedicated approaches such as the biotin-switch technique (BST, see below), over 3000 protein candidates for S-nitrosylation under normal and/or pathological conditions have been identified in animal cells (Hess and Stamler, 2012). A database (dbSNO 2.0) centralizing S-nitrosylated proteins collected from the literature is available at dbSNO 2.0 http://dbSNO.mbc.nctu.edu.tw (Chen et al., 2014). These proteins cover a wide range of cellular functions and include, amongst others, receptors, ion channels, signaling proteins, metabolic enzymes, proteases, chaperones, and structural proteins (as examples see Seth and Stamler, 2011; Nakamura et al., 2013; Ben-Lulu et al., 2014). Denitrosylation, the removal of NO from Cys residues, has also emerged as a key mechanism regulating protein activities, protein-protein interactions and more generally signaling (Martinez-Ruiz et al., 2013). For instance, certain proteins constitutively S-nitrosylated such as the pro-form of caspase 3 become activated upon denitrosylation (Mannick et al., 1999). This regulation also constitutes a powerful mechanism protecting cells from nitrosative stresses (Benhar et al., 2009). It involves non-enzymatical as well as enzymatical processes in which Trx and GSNO reductase catalyzing the reduction of GSNO, and therefore negatively regulating cellular levels of SNO, seem to play a major role (Figure 1) (Anand and Stamler, 2012; Martinez-Ruiz et al., 2013).

An ongoing issue concerns the selectivity and specificity of S-nitrosylation for certain proteins and Cys residues. Two major parameters have been highlighted. The first concerns the co-localization of target proteins with NOS. The presence of both the NO source and the S-nitrosylation substrate within discrete sub-cellular domains favors high local concentrations of nitrosylating species and the targeting of NO to specific Cys residues (Stamler et al., 2001; Mannick and Schonhoff, 2002; Kone et al., 2003; Martinez-Ruiz et al., 2011). This spatial and temporal confinement allows a tight regulation of NO signaling. The second is related to the reactivity of certain Cys residues. Structural, biophysical, biochemical, and bioinformatics approaches have been applied in order to define common features of the Cys residues of interest. The latter seem to be positioned in a favorable chemical context increasing their nucleophilicity and therefore thiol ionization (Lane et al., 2001). In this regard, the proximity of basic and aromatic residues emerged as a possible factor promoting S-nitrosylation (Derakhshan et al., 2007). This feature is not exclusive and other parameters have been pointed out such as the occurrence of an acid–base motif within 6–8 Å of the modified Cys (Doulias et al., 2010) or more distant but facilitating trans-nitrosylation (Marino and Gladyshev, 2010), the presence of hydrophobic residues allowing formation of efficient nitrosylating species in the vicinity of the Cys residue (Lane et al., 2001) and the localization of the Cys residue in a solvent-accessible surface of the protein (Marino and Gladyshev, 2012). The de-nitrosylation rate is probably another parameter of importance. In a recent study, Cheng et al. (2014) investigated the structural and biochemical features of S-nitrosylated Cys residues based on 213 structures of S-nitrosylated proteins for which structural information was available. According to previous studies, compared to non-S-nitrosylated Cys residues, the S-nitrosylated ones have a lower p*K_a_* and appeared to be more flexible and preferentially surrounded within 6 Å by basic residues which could enhance their deprotonation as well as stabilize their deprotonated state. A lower abundance of bulky residues (such as Phe, Tyr, Arg, and Leu) in the neighboring region within 8 Å of the Cys residues undergoing S-nitrosylation was also noted, suggesting that steric hindrance could be a disadvantage for the process of S-nitrosylation. Another feature listed in this study was a reduced frequency of Cys residues around the S-nitrosylation site, especially when the inter-residue distance was less than 3.5 Å. According to the authors, this structural particularity might reduce the competition for oxidant agents in the process of S-nitrosylation.

In sum, although partially conserved features of the nitrosylation sites emerged, these analyses have not yielded a dominant consensus motif. As pointed out by Smith and Marletta (2012), this statement is probably due to the various mechanisms leading to SNO formation. Web-servers dedicated to the prediction of S-nitrosylation sites have been developed. These programs are based on S-nitrosylation sites identified experimentally and, depending on the servers, features such as the identity of the flanking residues, solvent accessibility and the protein secondary and tertiary structures are taken into account for screening the Cys residue of interest. They are freely accessible: GPS-SNO (http://sno.biocuckoo.org/, Xue et al., 2010), SNOSite (http://csb.cse.yzu.edu.tw/SNOSite/, Lee et al., 2011), dbSNO 2.0 (http://dbSNO.mbc.nctu.edu.tw, Lee et al., 2012; Chen et al., 2014), iSNO-PseAAC (http://app.aporc.org/iSNO-PseAAC/, Xu et al., 2013), PSNO (http://59.73.198.144:8088/PSNO/, Zhang et al., 2014). Recently, Huang et al. (2014) proposed a new web-server (http://www.zhni.net/snopred/index.html) for the prediction of S-nitrosylation in which additional parameters such as evolutionary conservation and disorder status of amino-acid residues were also included.

## S-nitrosylation in plants: brief insights

The introduction of the BST described below has served as an impetus for screening for S-nitrosylated proteins in plants. Proteomic identification with this method was first achieved in plant tissues, cell suspensions or protein extracts exposed to nitrosylating agents, mainly GSNO. Additional studies also searched for proteins constitutively S-nitrosylated in plant cells or undergoing S-nitrosylation under physiological conditions including hormone signaling and responses to pathogens, PAMPs (Pathogen-Associated Molecular Patterns) and abiotic stresses such as high light, salinity, cold, and heavy metals (Spoel and Loake, 2011; Mengel et al., 2013; Romero-Puertas et al., 2013; Puyaubert and Baudouin, 2014; Trapet et al., 2014; Yu et al., 2014). Currently, well over a 100 proteins susceptible to S-nitrosylation have been identified. Amongst those, few have been thoroughly studied and confirmed to undergo S-nitrosylation *in vivo* (Astier et al., 2012b; Kovacs and Lindermayr, 2013; Skelly and Loake, 2013). Supplementary Table 1 lists several of these proteins and highlights the incidence of NO on their structure/function at the protein and, when investigated, at the physiological levels. What can we conclude about these pioneer studies? First, as reported in animals, S-nitrosylation appears to be implicated in the regulation of a wide array of protein functions and cell activities, particularly signaling, redox balance, metabolism, protein quality control and transcription. Second, the impact of this PTM differs according to the target protein: it promotes conformational changes, facilitates its oligomerization through the formation of disulfide linkages between monomers, inhibits the binding of cofactors such as ATP or NADPH or affects their activities by interacting with catalytic Cys residues. Third, enzymatic denitrosylation mechanisms occur through Trx and GSNO reductase (Malik et al., 2011; Kneeshaw et al., 2014).

In recent investigations, Kovacs and Lindermayr (2013) and Chaki et al. (2014) focused on the structural features of S-nitrosylation sites of plant proteins. For this purpose, the authors performed a computational prediction of S-nitrosylation sites of plant proteins experimentally found as S-nitrosylated and for which the corresponding Cys residue(s) have been identified by MS analysis. The programs GPS-SNO, SNOSites, and iSNO-PseAAC were used. The programs all predicted S-nitrosylation sites in those proteins. However, the number of Cys residues and their identity differed according to the computational programs. The GPS-SNO best matched the MS analysis. This performance confirmed a previous work demonstrating that amongst 485 potentially S-nitrosylated proteins collected from PubMed, the GPS-SNO program predicted at least one putative S-nitrosylation sites in 74% of them (Xue et al., 2010). Another in-depth analysis of the structural features of plant S-nitrosylated proteins was provided by Fares et al. (2011). Using the Motif-X-algorithm (http://motif-x.med.harvard.edu/) extracting overrepresented patterns from a sequence data set, these authors screened for motifs flanking S-nitrosylated Cys-residues (± 10 residues) amongst 53 proteins found to be constitutively regulated by this PTM in *Arabidopsis thaliana*. Three motifs involving hydrophobic residues were found (A-X(9)-C, C-X(6)-G, and C-X(2)-I). Interestingly, at least one of these motifs was detected in about half of 121 proteins that were previously identified as putatively S-nitrosylated in other studies. Amongst the selected proteins, 38 sites for S-nitrosylation were also predicted using the program SNOSite.

**Figure 2:**
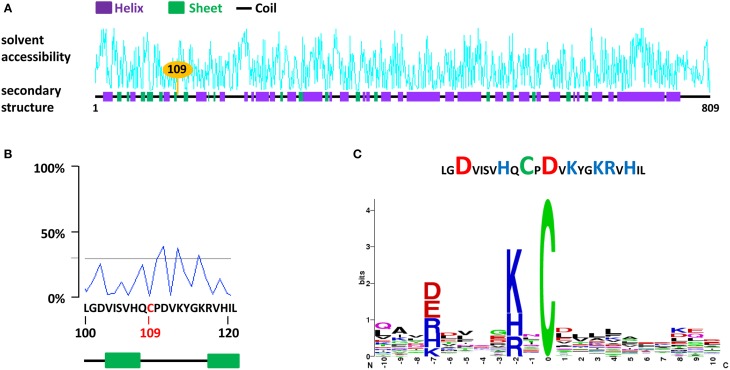
**A case study for the search for S-nitrosylation sites in the chaperone protein CDC48 of *A. thaliana* (AtCDC48). (A)** Analysis of AtCDC48 using dbSNO 2.0. The Cys-109, found to undergo S-nitrosylation *in vivo* (Fares et al., 2011) and *in vitro* (Astier et al., 2012a) is highlighted. **(B)** Details of the solvent accessibility and of the primary and secondary structure surrounding Cys-109. **(C)** Abundance of basic (blue) and acidic (red) residues flanking Cys-109 (upper panel) and statistically significant conserved motifs surrounding Cys-109 identified using the SNOsite web server (lower panel).

We applied this series of prediction to CDC48 (Cell Division Cycle 48), a chaperone-like AAA+ ATPase found to be constitutively S-nitrosylated in *A. thaliana* on Cys-109 (Fares et al., 2011) and on Cys-526 upon immune stimulation in tobacco (Astier et al., 2012a). In tobacco, Cys 110 (Cys 109 in *A. thaliana*), Cys-526 and Cys-664 were also found to undergo S-nitrosylation *in vitro* following the exposure of the recombinant protein with GSNO (Astier et al., 2012a). A case study for Cys-109 of *A. thaliana* CDC48 is shown Figure 2. In the primary sequence, Cys-109 is surrounded by two β sheets and its solvent accessibility appears to be low (Figures 2A,B). Furthermore, this residue locates in a region flanking with acidic and basic residues. Abundance of these residues around the Cys residues of interest is confirmed by the search for statistically significant conserved S-nitrosylation motifs (Figure 2C). Furthermore, as discussed above (Cheng et al., 2014), no Cys residue was found in the region flanking Cys-109.

## Identification of plant S-nitrosylated proteins: methodological aspects

### The biotin-switch technique

**Figure 3:**
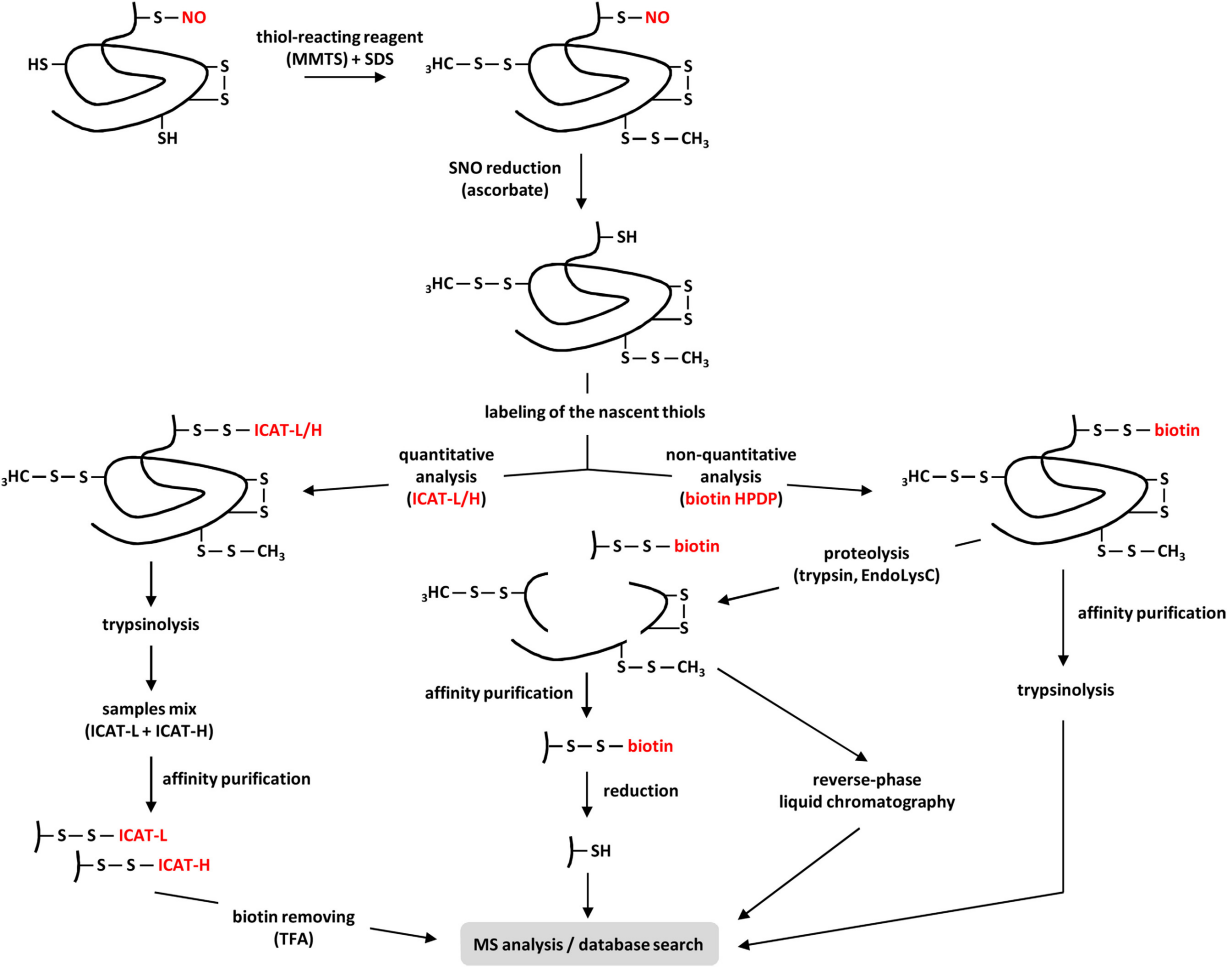
**Current proteomic strategies for the identification of S-nitrosylated proteins in plants**. After extraction from plant tissues or cell suspensions, proteins are subjected to the BST. Briefly, after methylthiolation of the free Cys-thiols with methyl-methane thiosulfonate (MMTS) in presence of sodium dodecyl sulfate (SDS), the S-NO bonds of S-nitrosylated proteins are selectively reduced by ascorbate. These steps can also be applied to purified recombinant proteins. Next, the resulting nascent thiols are labeled through two main approaches. In the non-quantitative approach, proteins are marked with a biotin-tagging reagent (usually biotin-HPDP). The resulting biotinylated proteins (e.g., initially S-nitrosylated) are purified by affinity, subjected to trypsinolysis before MS analysis. In order to selectively isolate the peptides undergoing S-nitrosylation, the biotinylated proteins are first digested by trypsin, Lys-C endoproteinase (EndoLysC) or both before purification by affinity or reverse-phase chromatography. When this latter approach is used, compared to their non-biotinylated counterparts, the biotinylated peptides show a mass shift of +428 Da in MS analysis. When the affinity purification is chosen, the captured biotinylated peptides are eluted by reduction using DTT or 2-mercaptoethanol before MS analysis. In the quantitative approach, nascent thiols are labeled with isotope-coded affinity tags (ICAT) containing 12C (ICAT-L for light) or 13C (ICAT-H for heavy) carbons, depending on the samples. After trypsinolysis, ICAT-L and ICAT-H samples are mixed before avidin purification. Finally, the biotin tags are cleaved by trifluoroacetic acid (TFA) and the 12C- and 13C-labeled peptides are subjected to MS quantitative/qualitative analysis.

The BST, initially developed by Jaffrey et al. (2001) (see also Jaffrey and Snyder, 2001) provides an efficient methodological tool for identifying S-nitrosylated proteins. In particular, this method greatly avoids the constraint of the inherent lability of protein S-NO groups. Basically, the BST involves three steps (Figure 3). In the first step, the blocking step, proteins extracted from tissues, cultured cells, purified organelles or recombinant proteins are incubated at 50°C with a thiol-reacting reagent, mainly methyl-methane thiosulfonate (MMTS), in the presence of sodium dodecyl sulfate (SDS). The combination of moderate heat and SDS favors protein denaturation and thus increases the accessibility of protein free thiols to the thiol-reacting reagent. This step allows the S-methylthiolation and therefore the blocking of free Cys thiols. In the second step, after removing the excess of MMTS, S-nitrosylated Cys residues are reduced by ascorbate to free Cys thiols. In this reaction, ascorbate acts as a nucleophile and undergoes a transnitrosation reaction to yield *O*-nitrosoascorbate which homolyzes into dehydroascorbate and NO (Holmes and Williams, 2000). As reported by Zhang et al. (2005), ascorbate is a poor reducing agent and long incubation times (up to 3 h) as well as high ascorbate concentrations (30 mM or more) significantly improve the sensitivity of the assay. In the third step, the nascent thiols (i.e., initially S-nitrosylated) are biotinylated with a sulfhydryl-specific biotinylating agent, mainly *N*-[6-(biotinamido)hexyl]-3'-(2'-pyridyldithio)propionamide (biotin–HPDP). Importantly, step 2 and 3 occur simultaneously to allow the immediate biotinylation of the newly liberated thiols. After removing the excess of ascorbate and biotin–HPDP, the resulting biotinylated proteins are enriched through classical purification processes using immobilized avidin or streptavidin and eluted by reduction (using 2-mercaptoethanol or dithiothreitol). Then, the purified proteins are further analyzed by western blotting or mass spectrometry (MS). A proteolytic step carried out with trypsin (Hao et al., 2006) or Lys-C endoproteinase (Astier et al., 2012a) or both (Morisse et al., 2014) can be introduced prior to avidin capture by affinity (Figure 3). The trypsin-based method is known as SNO-Site Identification (SNOSID, Hao et al., 2006). Besides optimizing the efficiency of the purification step, the main benefit of this strategy is to allow a selective isolation of the biotinylated peptides and therefore a better identification of the S-nitrosylation sites.

Although attractive, the BST is subject to artifacts. Notably, false positives can arise from incomplete blocking of free Cys thiols by the blocking reagent. Furthermore, as highlighted by Forrester et al. (2007), in the presence of indirect sunlight (for instance from a laboratory window), ascorbate reduces biotin-HPDP to biotin-SH, which in turn could lead to artifactual protein biotinylation *via* thiol/disulfide exchange with methylthiolated Cys residues resulting from the first step. Protection from sunlight (e.g., working in darkness) eliminates this risk of false positives. Omission of MMTS, ascorbate and biotin-HPDP are also used as controls. Furthermore, elimination of SNO by photolysis through a strong ultraviolet light source before the application of the BST is recommended as another control for the specificity of the assay (Forrester et al., 2007).

Variants of the BST developed by Jaffrey et al. (2001), including quantitative approaches and the use of protein microarrays have been reported and successfully used (Torta et al., 2008; Astier et al., 2011; Seth and Stamler, 2011; Wang and Xian, 2011; Lee et al., 2014). For instance, Qu et al. (2014) developed a modified BST in which the nascent thiols resulting from ascorbate reduction of the SNO bonds are irreversibly labeled with an isobaric iodoacetyl tandem mass tag (iodoTMT) reagent. The iodoTMT consists of a thiol-reactive iodoacetyl group, a MS-neutral spacer arm and a tandem mass spectrometry (MS/MS) reporter with a mass ranging from 126 (iodoTMT-126) to 131 (iodoTMT-131). When comparing protein samples, it is therefore possible to label each sample with a specific iodoTMT. Then, the differently labeled protein samples are pooled, trypsin-digested and enriched using anti-TMT antibody resin. During MS/MS analysis, the MS/MS reporters are cleaved, thus generating reporter ions with unique m/z (of 126–131). Therefore, this strategy provides an accurate quantification of the S-nitrosylated proteins. Beside such approaches, others have been designed for capturing S-nitrosylated proteins (reviewed by Raju et al., 2012) such as the Trx trapping strategy (Ben-Lulu et al., 2014). This latter method is based on the denitrosylase activity of Trx. The denitrosylation enzymatic process involves the formation of a transient intermolecular disulfide bond between Trx and its S-nitrosylated substrate. Then, thanks to an intramolecular attack mediated by a Trx Cys residue (named the resolving Cys), the denitrosylated target and the oxidized Trx are released. In the Trx trapping strategy, a mutated Trx in which the resolving Cys was mutated is generated in order to stabilize the transient intermolecular disulfide bond between Trx and the S-nitrosylated substrate. In Ben-Lulu et al. (2014) investigation, cell lysates of murine macrophages or human monocytes exposed to lipopolysaccharides/interferon-γ or exogenous NO, respectively, were incubated with the Trx mutant. Subsequently, the proteins captured by Trx were pulled down and released with DTT before identification by MS analysis. Hundreds of S-nitrosylated proteins were next identified including novel candidates playing key functions in cellular homeostasis and signaling.

In plants, identification of S-nitrosylated proteins was based primarily on the initial BST with a few adjustments. Recently, Fares et al. (2011) and Puyaubert et al. (2014) combined the BST with isotope-coded affinity tags (ICAT) as substitutes for biotin-HPDP to provide a quantitative analysis of proteins constitutively S-nitrosylated or undergoing this PTM in *A. thaliana* cell suspensions facing abiotic stresses. ICAT is based on an iodoacetamide group, which reacts specifically with cysteine thiols, connected to biotin by a linker that contains 12C (light: ICAT-L) or 13C (heavy: ICAT-H) carbons, thus differing in mass by 9 Da. It also contains an acid-cleavable group allowing the removal of the biotin by treating the labeled peptides with trifluoroacetic acid (TFA). In this procedure first reported by Wu et al. (2011), newly exposed free thiols (i.e., initially S-nitrosylated) resulting from ascorbate reduction were differentially labeled with either ICAT-L or ICAT-H (Figure 3). More precisely, protein samples extracted from stressed cells were labeled with ICAT-H, while untreated samples resulting from untreated cells were labeled with ICAT-L. After trypsin digestion, ICAT-H- and ICAT-L-labeled peptides were mixed before avidin purification. Finally, the biotin tag was cleaved using TFA in order to generate higher quality MS/MS spectra for the identification and quantification of SNO-peptides.

### Mass spectrometry

Mass spectrometry involves the analysis of ionized molecules with the purpose to determine their structure, molecular weight and abundance. In the biological field, two ionization techniques are mainly used: MALDI and electrospray ionization (ESI). Karas and Hillenkamp (1988) were the first to demonstrate the ability of MALDI to detect molecules in a sub-nanogram range. Fenn et al. (1989) were also able to achieve similar results in the detection of large molecules using ESI. Since the publication of their results, MS has become a powerful analytical tool in a large range of biological investigations. In principle, the workflow of MS consists of ionization of a sample in an ion source, separation of the ionized molecules according to their mass-to-charge ratio in an analyzer, detection of the ionized molecules in a detector and generation of a mass spectrum. Although both MALDI and ESI allow proteins or peptides to be ionized with high sensitivity, important differences exist between both techniques (reviewed in Silva et al., 2013; Chicooree et al., 2014). One of note is related to the insensitivity of MALDI to salt contaminants. This feature makes it a preferred option for analyzing peptides recovered from electrophoresis gels by peptide mass fingerprint (Henzel et al., 1993). Typically, modified protein samples may be separated using SDS-PAGE, digested by a sequence specific protease and analyzed in a mass spectrometer connected to a MALDI or ESI ionization source. The peptides resulting from digestion can be further separated by liquid chromatography in order to facilitate identification. Relying on the mass-to-charge shift of a specific PTM, mass spectrometry can be successfully used to detect and identify the modification. However, for the unequivocal assignment of a given modification site, MS/MS experiments are required to prove that the mass shift detected in the precursor ion is also observed in the fragment ions carrying the modified amino acid residue.

S-nitrosylation sites in plant proteins can be identified by MS using approaches with some differences. For instance, the addition of the NO group molecule to a Cys residue increases its mass to +29 Da. This mass shift can be detected with the proper instrument optimization and sample preparation. However, with the MALDI source the peptides bearing this mass addition are decomposed upon laser ionization, which makes their identification difficult (Kaneko and Wada, 2003). The ESI source is most commonly used to probe this mass increase and has been addressed in some recent reviews (Foster, 2012; Chen et al., 2013; Devarie-Baez et al., 2013; Chicooree et al., 2014). For instance, peroxiredoxin II E (PrxII E) was shown to undergo S-nitrosylation in *A. thaliana* leaves infected with an avirulent strain of the bacterial pathogen *Pseudomonas syringae* pv. *tomato* (Romero-Puertas et al., 2008). Using ESI-MS/MS, the same team was able to measure the characteristic mass shift of +29 Da of a Cys-containing peptide of PrxIIE following its exposure to GSNO (Romero-Puertas et al., 2007). For this purpose, two experiments were performed. In the first one, the recombinant PrXIIE protein was treated with GSNO and then processed with trypsin and AspN protease before MS analysis. In the second one, the recombinant protein was first digested and the resulting peptide mixture was treated with the NO donor before MS analysis. As reported by the authors, treating the peptide mixture with GSNO instead of the full-length protein reduced the time between NO donor treatment and MS analysis from about 4 h to 15 min, increasing the yield of detection of the labile SNO modification. On the other hand, S-nitrosylated Cys residues can also be identified once biotinylated during application of the BST. The resulting peptides can either be separated by reverse-phase liquid chromatography, enriched and analyzed by MALDI-TOF-MS in order to detect the addition of 428 Da (Astier et al., 2012a) or, as discussed previously, captured on immobilized avidin and selectively released from avidin by reduction of the disulfide linker (Hao et al., 2006) (Figure 3). This latter approach presents some drawbacks as labeled peptides are reduced before MS analysis. Consequently, it is not possible, for peptides with multiple Cys residues, to precisely determine the S-nitrosylation site. Besides, non-S-nitrosylated peptides can also be disulfide-bonded to a biotin-labeled peptide, which after reduction may be mistaken as S-nitrosylated. This problem was solved by using acidic conditions to release the biotinylated peptides from resin without the loss of the biotin linker, facilitating thus the identification of the specific site containing the additional mass (Greco et al., 2006). More generally, the number of reports on the application of MS for the identification of S-nitrosylated sites in plant proteins has steadily increased in the last years. With few exceptions, all are based on the BST and differ only in the MS equipment utilized, ion sources and the use of liquid chromatography.

## Conclusion

The continued success of NO research in plants depends in part on the identification and functional characterization of S-nitrosylated proteins. Such investigation is of importance to advance our understanding of the regulation and organization of the NO-dependent cellular pathways and networks regulating key physiological processes.

During the last few years, there has been an important investment to decipher S-nitroso-proteomes of plant tissues or cell suspensions. BST has been and is still the most commonly used technique for this purpose. Compared to the original protocol described by Jaffrey et al. (2001), few modifications have been made such as a tighter control of ascorbate concentration or the use of thiol-reacting reagents other than MMTS. Undoubtedly, this approach provided significant results and opened new roads of research. Importantly, BST also generates false positives and a thorough analysis of such limitation is still lacking. Furthermore, BST does not allow a precise location of the Cys residue of interest on peptides with multiple Cys residues by MS analysis. Highlighting these drawbacks, for most of the candidates identified so far, a physiological role has not been ascribed to S-nitrosylation. However, at our current level of knowledge, the approach of studying S-nitrosylation by focusing on single proteins is essential and remains the most efficient way to decipher the complexity of the molecular mechanisms inherent to NO physiological function in plants. Therefore, while the list of plant protein candidates for S-nitrosylation is increasing, there is a need to identify the particular Cys residue(s) modified and to define the physiological relevance of their S-nitrosylation. Identification of the Cys residues of interest is also required to further characterize the physico-chemical, biochemical and structural features of S-nitrosylation sites. In addition, this approach will enrich the current databases centralizing S-nitrosylated proteins and, consequently, will help in defining parameters that must be fed into computational prediction of S-nitrosylation sites. Such combinations of proteomic-scale approaches with bioinformatics tools could therefore hold great promise for the elucidation of NO functions in plant cells.

Another issue concerns the quantitative aspects of the BST. So far, few studies provided a quantitative analysis of S-nitrosylated plant proteins. This might be partly explained by the fact that in most of the studies published so far, S-nitrosylation has been investigated through the use of NO donors delivering doses of NO beyond the range of physiological concentrations. Also, such approaches do not take into account the spatial and temporal features of NO-induced PTM. Indeed, it should be emphasized that under physiological contexts, S-nitrosylation is a dynamic and labile PTM restricted to a small subset of proteins which might be located in discrete subcellular compartments. Although useful, NO donors poorly mimic such biological conditions. We assume that quantitative analysis should provide a greater appreciation of this process *in vivo* and a better view of the S-nitrosylation states of proteins, in particular when comparing various conditions. In this regard, the use of isobaric iodoTMT reagents allowing the simultaneous comparison of several samples in a single MS/MS analysis (Qu et al., 2014) looks promising.

**Figure 4:**
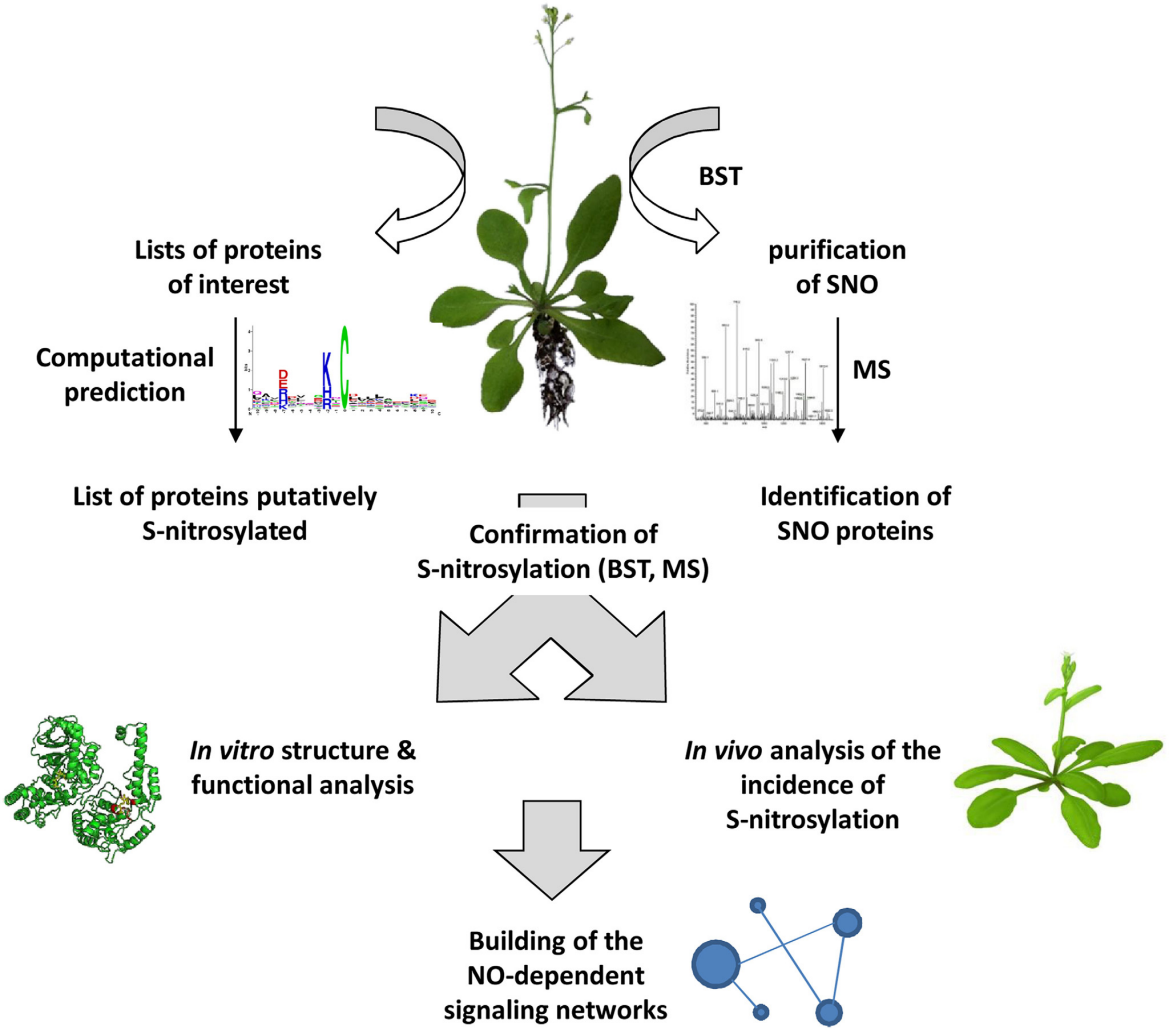
**General strategy for investigating S-nitrosylation-based processes in plants**. S-nitrosylated proteins (SNO) could be purified and identified from plant tissues using the BST and MS analysis. Alternatively, a search of proteins putatively S-nitrosylated could be undertaken from lists of proteins involved in particular physiological processes using dedicated web-servers. All the subsequent steps will help in building NO-dependent signaling networks and also will provide new information completing SNO databases and web servers dedicated to SNO sites.

In animal biology, the burgeoning of new technologies such as high-density protein microarray chips, and the improvement of established approaches such as BST and combined MS analysis have contributed to progress in the field of NO research. Plant biologists need to better consider these technological aspects. New tools and techniques are indeed required to provide both quantitative and qualitative data allowing a detailed and integrated insight into S-nitrosylation of plant proteins (Figure 4). Accompanying strategies include mutagenesis, structural analysis, subcellular localization, functional genomics and the search of interacting partners. This latter approach is of prime importance as certain S-nitrosylated proteins were shown to belong to protein complexes considered as fundamental building blocks of cellular signaling.

### Conflict of interest statement

The authors declare that the research was conducted in the absence of any commercial or financial relationships that could be construed as a potential conflict of interest.
