# Supplementary material for: Protein S-nitrosylation: specificity and identification strategies in plants
Source: Front Chem. 2015 Jan 7;2:114. doi: 10.3389/fchem.2014.00114 (PMC4285867; doi:10.3389/fchem.2014.00114)
Supplement: Supplementary file 1 [file Table1.PDF]

Supplementary Table 1 | Examples of plant proteins regulated through S-nitrosylation

Protein	Plant species	S-nitrosylated Cys residue(e)	Impact of S-nitrosylation	Reference
AHP1	*A. thaliana*	115	- Represses phosphorylation of AHP1<br>- Represses cytokinin signaling	(Feng et al., 2013)
APX	*Nicotiana tabacum,*<br>*A. thaliana*<br>*Pisum sativum*	32, 168?	- Enhancement of activity, partial inhibition through denitrosylation<br><br>- Inhibition of activity in response to cell death inducers<br>- Might promote APX degradation through the ubiquitin-proteasome pathway	(Correa-Aragunde et al., 2013)<br>(Begara-Morales et al., 2014)<br>(de Pinto et al., 2013)
CDC48	*Nicotiana tabacum,*<br><br>*A. thaliana*	*In vivo*: 110, 526<br>*In vitro*: 110, 526, 664<br><br>109	Inhibition of ATPase activity through steric hindrance interfering with ATP binding<br><br>Residue constitutively S-nitrosylated	(Astier et al., 2012)<br><br>(Fares et al., 2011)
GAPDH	*Nicotiana tabacum*<br>*A. thaliana*	?<br>149	- Inhibition of activity<br>- Inhibition of activity	(Wawer et al., 2010)<br>(Lindermayr et al., 2005)<br>(Zaffagnini et al., 2013)
GSNOR	*A. thaliana*	?	- Inhibition of activity<br>- Regulation of the nitrogen assimilation pathway	(Frungillo et al., 2014)
MC9	*A. thaliana*	147	Keeps AtMC9 in its inactive unprocessed zymogenic form	(Belenghi et al., 2007)
MYB2<br>MYB30	*A. thaliana*	53 (MYB2)<br>49 ?, 53 ? (MYB30)	- Inhibition of DNA binding<br>- Modification of the secondary structure	(Serpa et al., 2007)<br>(Tavares et al., 2014)
NRP1	*A. thaliana*	156	- Inhibition by promoting the oligomerization of NPR1 monomers through disulfide linkages<br>- Induces NPR1 nuclear translocation	(Tada et al., 2008)<br><br>(Lindermayr et al., 2010)
PrxII E	*A. thaliana*	121	- Inhibition of peroxynitrite reductase activity<br>- Peroxynitrite accumulation, increased cellular tyrosine nitration	(Romero-Puertas et al., 2007)
RBOHD	*A. thaliana*	890	- Inhibition of $O_2^{\bullet -}$ synthesis through destabilization of FAD binding<br>- Negative regulation of cell death	(Yun et al., 2011)

			propagation during immune responses	
SABP3	*A. thaliana*	280	- Inhibition of enzymatic activity<br>- Inhibition of salicylic acid binding	(Wang et al., 2009)
TGA1	*A. thaliana*	172, 287	- Activation of transcriptional activity through binding to defense gene promoters	(Lindermayr et al., 2010)
TIR1	*A. thaliana*	140 (?), 480 ?	- Might promote the interaction of TIR1 with transcriptional repressors of auxin signaling and their subsequent degradation	(Terrile et al., 2012)

AHP1: Phosphotransfer Protein 1, APX: Ascorbate Peroxidase, CDC48: Cell Division Cycle 48, GAPDH: glyceraldehyde-3-phosphate dehydrogenase, GSNOR: GSNO reducatase, MC9: Metacaspase 9, MYB2 and MYB30: transcription factors of the MYB family, NPR1: nonexpressor of pathogenesis-related gene 1, PrxII E: Peroxiredoxin II E, RBOHD: Respiratory Burst Oxidase Homolog D (NADPH oxidase), SABP3: Salicylic Acid Binding Protein 3 (carbonic anhydrase), TGA1: transcription factor of the TGA family, TIR1: Transport Inhibitor Response 1.

REFERENCES

Astier, J., Besson-Bard, A., Lamotte, O., Bertoldo, J., Bourque, S., Terenzi, H., and Wendehenne, D. (2012). Nitric oxide inhibits the ATPase activity of the chaperone-like AAA+ ATPase CDC48, a target for S-nitrosylation in cryptogein signalling in tobacco cells. *Biochem J* 447**,** 249-260. doi: 10.1042/BJ20120257.

Begara-Morales, J.C., Sanchez-Calvo, B., Chaki, M., Valderrama, R., Mata-Perez, C., Lopez-Jaramillo, J., Padilla, M.N., Carreras, A., Corpas, F.J., and Barroso, J.B. (2014). Dual regulation of cytosolic ascorbate peroxidase (APX) by tyrosine nitration and S-nitrosylation. *J Exp Bot* 65**,** 527-538. doi: 10.1093/jxb/ert396.

Belenghi, B., Romero-Puertas, M.C., Vercammen, D., Brackenier, A., Inze, D., Delledonne, M., and Van Breusegem, F. (2007). Metacaspase activity of Arabidopsis thaliana is regulated by S-nitrosylation of a critical cysteine residue. *J Biol Chem* 282**,** 1352-1358. doi: 10.1074/jbc.M608931200.

Correa-Aragunde, N., Foresi, N., Delledonne, M., and Lamattina, L. (2013). Auxin induces redox regulation of ascorbate peroxidase 1 activity by S-nitrosylation/denitrosylation balance resulting in changes of root growth pattern in Arabidopsis. *J Exp Bot* 64**,** 3339-3349. doi: 10.1093/jxb/ert172.

De Pinto, M.C., Locato, V., Sgobba, A., Romero-Puertas Mdel, C., Gadaleta, C., Delledonne, M., and De Gara, L. (2013). S-nitrosylation of ascorbate peroxidase is part of programmed cell death signaling in tobacco Bright Yellow-2 cells. *Plant Physiol* 163**,** 1766-1775. doi: 10.1104/pp.113.222703.

Fares, A., Rossignol, M., and Peltier, J.B. (2011). Proteomics investigation of endogenous S-nitrosylation in Arabidopsis. *Biochem Biophys Res Commun* 416**,** 331-336. doi: 10.1016/j.bbrc.2011.11.036.

Feng, J., Wang, C., Chen, Q., Chen, H., Ren, B., Li, X., and Zuo, J. (2013). S-nitrosylation of phosphotransfer proteins represses cytokinin signaling. *Nat Commun* 4**,** 1529. doi: 10.1038/ncomms2541.

Frungillo, L., Skelly, M.J., Loake, G.J., Spoel, S.H., and Salgado, I. (2014). S-nitrosothiols regulate nitric oxide production and storage in plants through the nitrogen assimilation pathway. *Nat Commun* 5**,** 5401. doi: 10.1038/ncomms6401.

Lindermayr, C., Saalbach, G., and Durner, J. (2005). Proteomic identification of S-nitrosylated proteins in Arabidopsis. *Plant Physiol* 137**,** 921-930. doi: 10.1104/pp.104.058719.

Lindermayr, C., Sell, S., Muller, B., Leister, D., and Durner, J. (2010). Redox regulation of the NPR1-TGA1 system of Arabidopsis thaliana by nitric oxide. *Plant Cell* 22**,** 2894-2907. doi: 10.1105/tpc.109.066464.

Romero-Puertas, M.C., Laxa, M., Matte, A., Zaninotto, F., Finkemeier, I., Jones, A.M., Perazzolli, M., Vandelle, E., Dietz, K.J., and Delledonne, M. (2007). S-nitrosylation of peroxiredoxin II E promotes peroxynitrite-mediated tyrosine nitration. *Plant Cell* 19**,** 4120-4130. doi: 10.1105/tpc.107.055061.

Serpa, V., Vernal, J., Lamattina, L., Grotewold, E., Cassia, R., and Terenzi, H. (2007). Inhibition of AtMYB2 DNA-binding by nitric oxide involves cysteine S-nitrosylation. *Biochem Biophys Res Commun* 361**,** 1048-1053. doi: 10.1016/j.bbrc.2007.07.133.

Tada, Y., Spoel, S.H., Pajerowska-Mukhtar, K., Mou, Z., Song, J., Wang, C., Zuo, J., and Dong, X. (2008). Plant immunity requires conformational changes [corrected] of NPR1 via S-nitrosylation and thioredoxins. *Science* 321**,** 952-956. doi: 10.1126/science.1156970.

Tavares, C.P., Vernal, J., Delena, R.A., Lamattina, L., Cassia, R., and Terenzi, H. (2014). S-nitrosylation influences the structure and DNA binding activity of AtMYB30 transcription factor from Arabidopsis thaliana. *Biochim Biophys Acta* 1844**,** 810-817. doi: 10.1016/j.bbapap.2014.02.015.

Terrile, M.C., Paris, R., Calderon-Villalobos, L.I., Iglesias, M.J., Lamattina, L., Estelle, M., and Casalongue, C.A. (2012). Nitric oxide influences auxin signaling through S-nitrosylation of the Arabidopsis TRANSPORT INHIBITOR RESPONSE 1 auxin receptor. *Plant J* 70**,** 492-500. doi: 10.1111/j.1365-313X.2011.04885.x.

Wang, Y.Q., Feechan, A., Yun, B.W., Shafiei, R., Hofmann, A., Taylor, P., Xue, P., Yang, F.Q., Xie, Z.S., Pallas, J.A., Chu, C.C., and Loake, G.J. (2009). S-nitrosylation of AtSABP3 antagonizes the expression of plant immunity. *J Biol Chem* 284**,** 2131-2137. doi: 10.1074/jbc.M806782200.

Wawer, I., Bucholc, M., Astier, J., Anielska-Mazur, A., Dahan, J., Kulik, A., Wyslouch-Cieszynska, A., Zareba-Koziol, M., Krzywinska, E., Dadlez, M., Dobrowolska, G., and Wendehenne, D. (2010). Regulation of Nicotiana tabacum osmotic stress-activated protein kinase and its cellular partner GAPDH by nitric oxide in response to salinity. *Biochem J* 429**,** 73-83. doi: 10.1042/BJ20100492.

Yun, B.W., Feechan, A., Yin, M., Saidi, N.B., Le Bihan, T., Yu, M., Moore, J.W., Kang, J.G., Kwon, E., Spoel, S.H., Pallas, J.A., and Loake, G.J. (2011). S-nitrosylation of NADPH oxidase regulates cell death in plant immunity. *Nature* 478**,** 264-268. doi: 10.1038/nature10427.

Zaffagnini, M., Morisse, S., Bedhomme, M., Marchand, C.H., Festa, M., Rouhier, N., Lemaire, S.D., and Trost, P. (2013). Mechanisms of nitrosylation and denitrosylation of cytoplasmic glyceraldehyde-3-phosphate dehydrogenase from Arabidopsis thaliana. *J Biol Chem* 288**,** 22777-22789. doi: 10.1074/jbc.M113.475467.
